# Dorsal midline ectopic mammary tissue as a cutaneous marker of spinal dysraphism: intradural lipoma and tethered cord in a child with literature review

**DOI:** 10.1007/s00381-026-07350-5

**Published:** 2026-06-18

**Authors:** Paula Alcazar, Hélène Person, Federico Di Rocco, Lisa Boudy, Camilla de Laurentis, Cécile Picard, Alexandre Vasiljevic, Alexandru Szathmari, Pierre-Aurélien Beuriat

**Affiliations:** 1 Paediatric Neurosurgery Unit, Hôpital Femme Mère Enfant, Hospices Civils de Lyon, Bron, France; 2 Paediatric Plastic Surgery Unit, Hôpital Femme Mère Enfant, Hospices Civils de Lyon, Bron, France; 3 Neuropathology and Foetology Unit, Centre de Pathologie et Neuropathologie Est, Hospices Civils de Lyon, Bron, France

**Keywords:** Spinal cord tethering, Cutaneous markers, Pediatric neurosurgery, Ectopic breast tissue

## Abstract

**Purpose:**

Supernumerary breasts (polymastia) and supernumerary nipples (polythelia) are congenital anomalies resulting from incomplete regression of the embryonic mammary ridge. They most frequently occur along the embryologic milk line extending from the axilla to the groin. Ectopic localization outside this line is rare, and dorsal presentations are exceptional. In such cases, an association with underlying spinal dysraphism has occasionally been reported.

**Methods:**

We report the case of an 11-year-old girl presenting with a congenital dorsal midline swelling initially diagnosed as a lipoma. Progressive enlargement and the appearance of a central pigmented structure prompted further evaluation. Clinical examination confirmed a dorsal midline areola-nipple complex with underlying tumefaction. Spinal MRI demonstrated an intradural lipoma at the T4 level associated with a fibrous tract extending toward the cutaneous lesion, resulting in a tethered cord configuration.

**Results:**

The patient underwent combined resection of the ectopic mammary tissue and neurosurgical detethering with partial lipoma resection. Postoperative recovery was uneventful with no neurological deficit.

**Conclusion:**

Dorsal midline ectopic mammary tissue is extremely rare and may represent a cutaneous marker underlying spinal dysraphism. Careful clinical examination and spinal imaging should therefore be considered in such atypical presentations.

## Introduction

Cutaneous stigmata are important clinical indicators of occult spinal dysraphism in children. While lipomas, dermal sinus tracts, and vascular lesions are well-recognized markers, ectopic mammary structures in the dorsal midline have only rarely been reported.

Supernumerary mammary structures arise from incomplete regression of the embryonic mammary ridge, also known as the milk line [1]. This ectodermal structure develops around the sixth week of gestation, extending bilaterally from the axillary region to the inguinal area [2]. Normally, the ridge regresses except at the pectoral region, where the definitive mammary glands develop.

Failure of this regression may lead to ectopic mammary tissue, which may range from isolated nipples (polythelia) to complete breast tissue with glandular components (polymastia) [2, 3].

## Historical background

The reported prevalence of supernumerary nipples is approximately 0.2–5% of the population, with most lesions located along the embryologic milk line [4].

While supernumerary nipples and accessory breast tissue most commonly occur along this line, ectopic mammary tissue outside the milk line is rare and has been reported in unusual locations such as the face, shoulder, thigh, and back [2, 5].

Though cutaneous markers are important indicators of occult spinal dysraphism, ectopic mammary tissue has rarely been reported in such a context. We describe a rare dorsal midline ectopic mammary tissue associated with an intradural lipoma and a tethered cord in a child. This case emphasizes the diagnostic value of spinal MRI in atypical congenital dorsal lesions.

## Clinical presentation

**Fig. 1:**
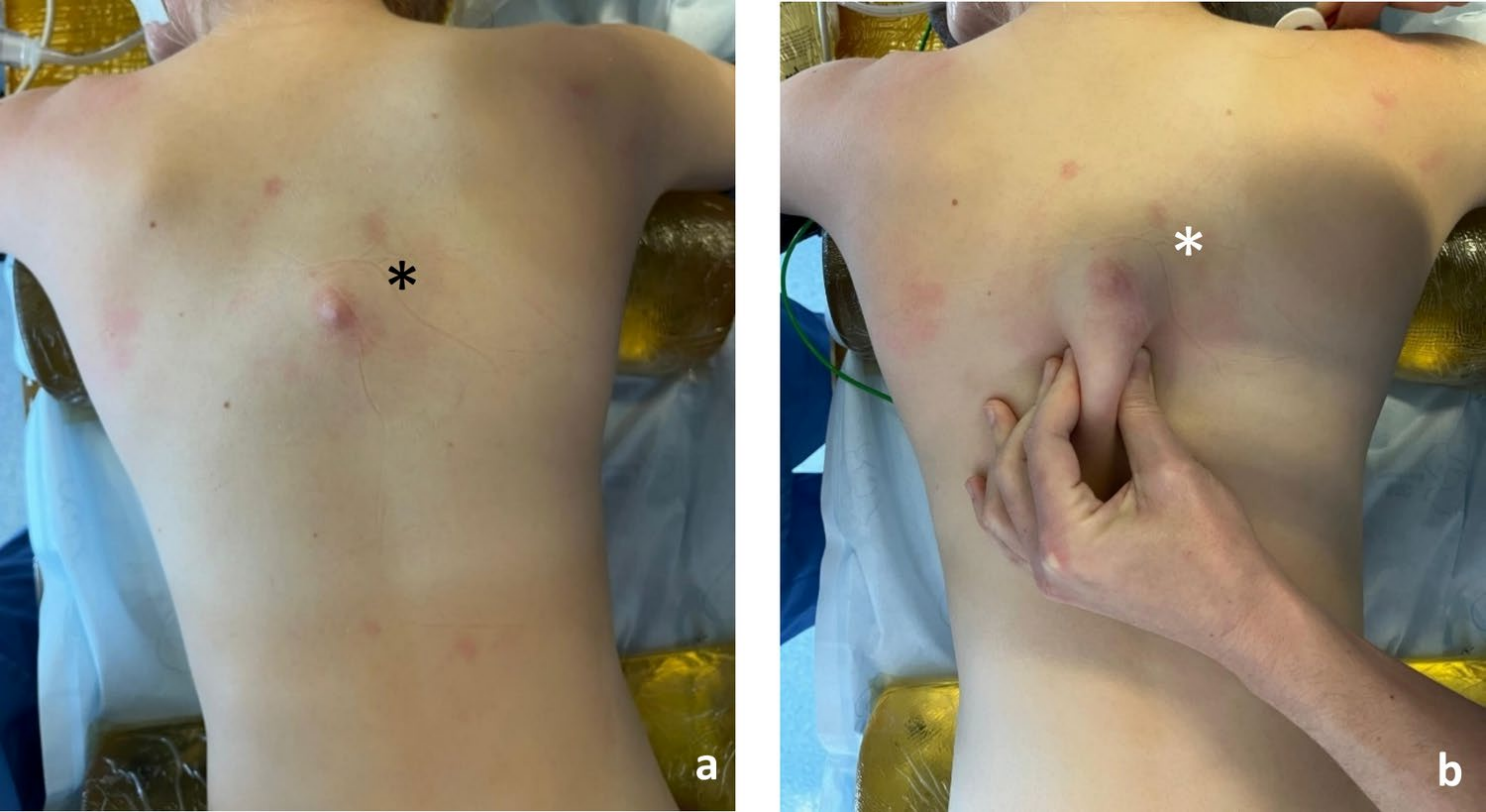
**a** Clinical appearance of the lesion (black *asterisk*). **b** Dorsal midline cutaneous lesion demonstrating a small areola-nipple complex associated with a subcutaneous tumefaction measuring approximately 5 cm (white *asterisk*)

An 11-year-old girl presented with a dorsal swelling that had been present since birth but had progressively enlarged (Fig. 1a, b). Initial ultrasound performed earlier in childhood had suggested a subcutaneous lipoma. Over time, a central pigmented area resembling a nipple gradually appeared.

## Diagnosis

Ultrasound performed at the age of 11 demonstrated hypertrophy of the dorsal subcutaneous soft tissues measuring 5 × 4 cm, centered by a 9-mm hypoechoic dendritic mammary bud located posterior to a central nipple-like structure, suggestive of ectopic mammary tissue.

Clinical examination revealed a small dorsal midline areola-nipple complex associated with a subcutaneous tumefaction measuring approximately 5 × 5 cm (Fig. 1a, b). Pubertal breast development had begun (Tanner S3). No additional thoraco-abdominal or dorsal cutaneous lesions were identified, and no spinal deformity or palpable vertebral irregularity was noted.

**Fig. 2:**
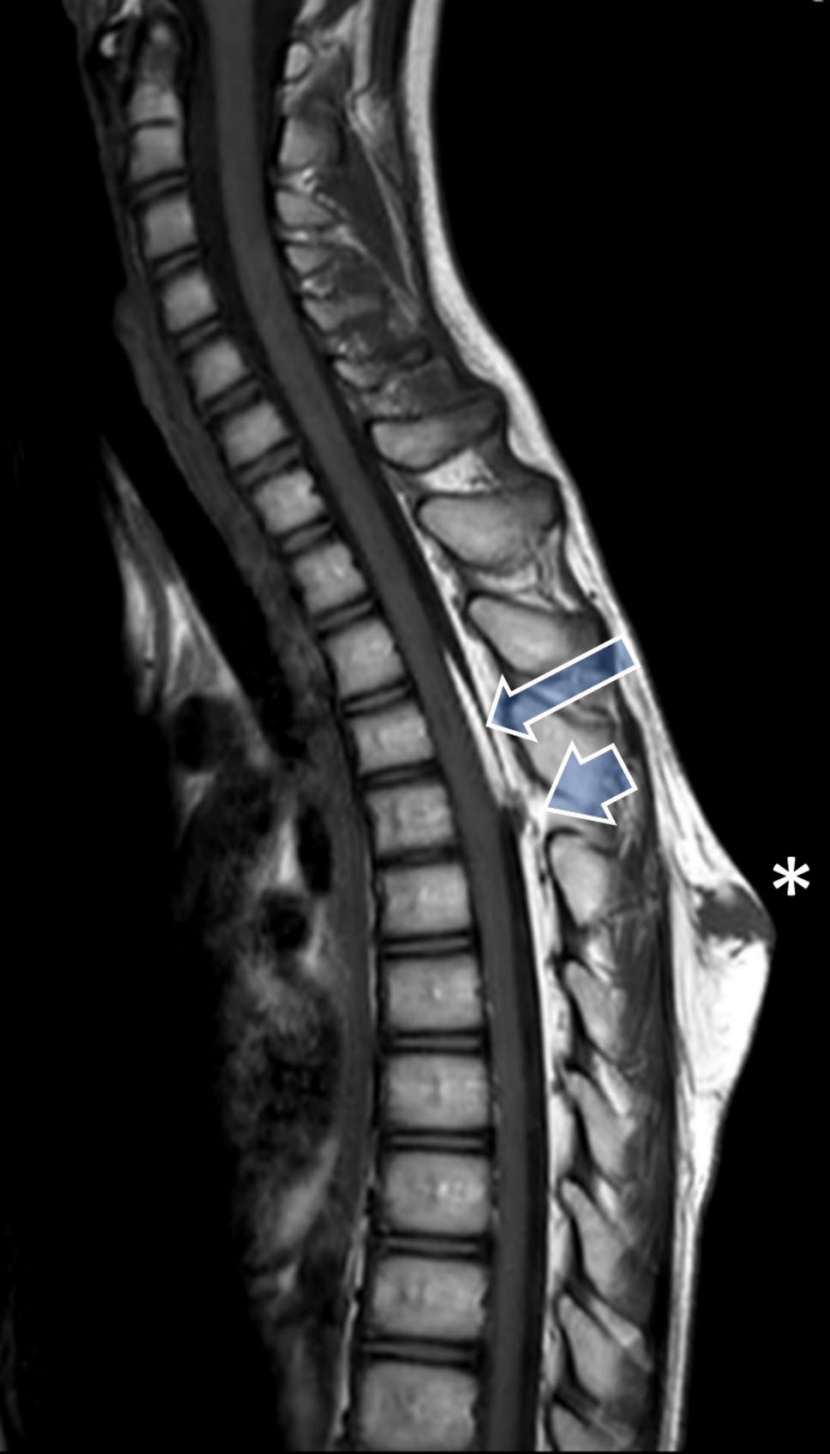
Spinal MRI findings: Sagittal T1-weighted MRI showing an intradural lipoma (*long arrow*) at the level T4 associated with a fibrous tract (*short arrow*) extending from the spinal canal toward the dorsal cutaneous lesion (*asterisk*), producing a tethered cord configuration

Given the unusual midline location, spinal magnetic resonance imaging (MRI) was performed. Imaging revealed an intradural lipoma at the T4 level associated with a fibrous tract extending from the spinal canal to the dorsal cutaneous lesion, producing an appearance consistent with spinal tethered cord (Fig. 2).

## Management

The case was discussed in a multidisciplinary meeting including plastic surgeons and neurosurgeons, and prophylactic surgical detethering combined with excision of the ectopic mammary tissue was recommended.

Under general anaesthesia, the patient was placed in prone position (Fig. 1a, b). A fusiform incision encompassing the areola-nipple complex and extending superiorly was designed.

**Fig. 3:**
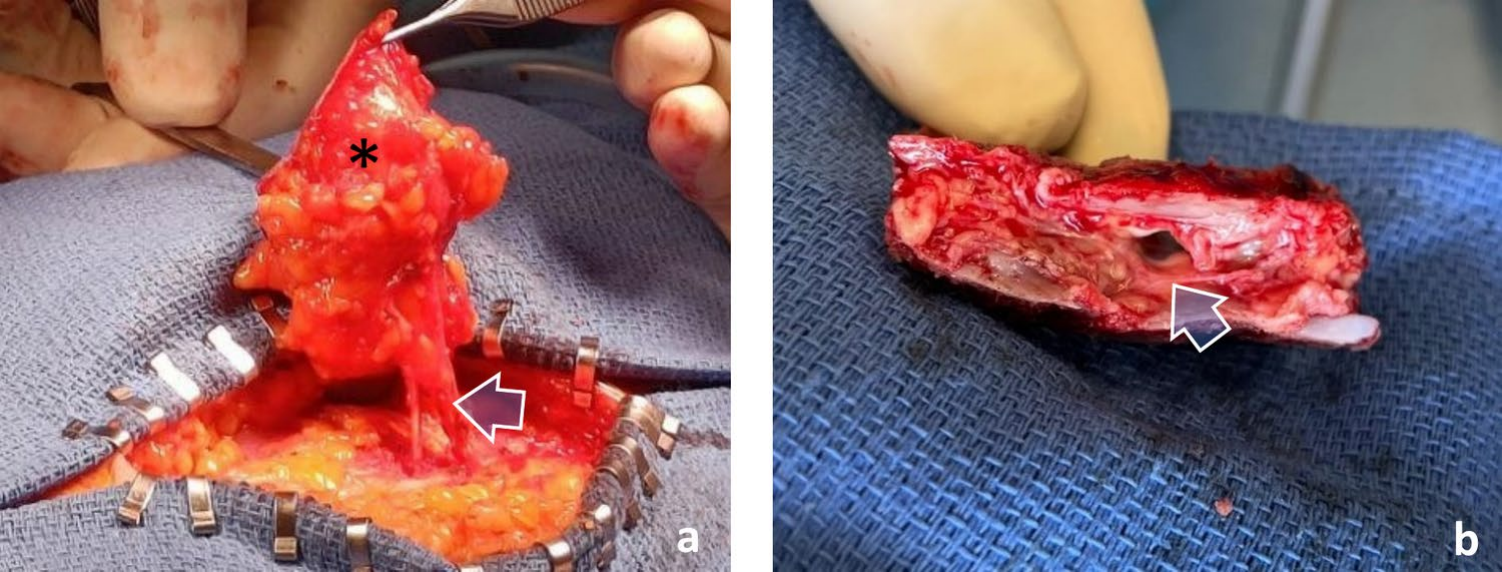
Intraoperative view of the fibrous tract. **a** Surgical exposure demonstrating the fibrous tract (*arrow*) connecting the dorsal lesion (*asterisk*) to the spinal elements after subperiosteal dissection of the spinous processes. **b** Fusion of the laminae at the T4 level with the canal of the fibrous tract (*arrow*)

Excision of the lesion was performed, including the mammary gland and surrounding adipose tissue. Circumferential dissection identified a fibrous tract extending toward the spinal canal (Fig. 3a, b).

**Fig. 4:**
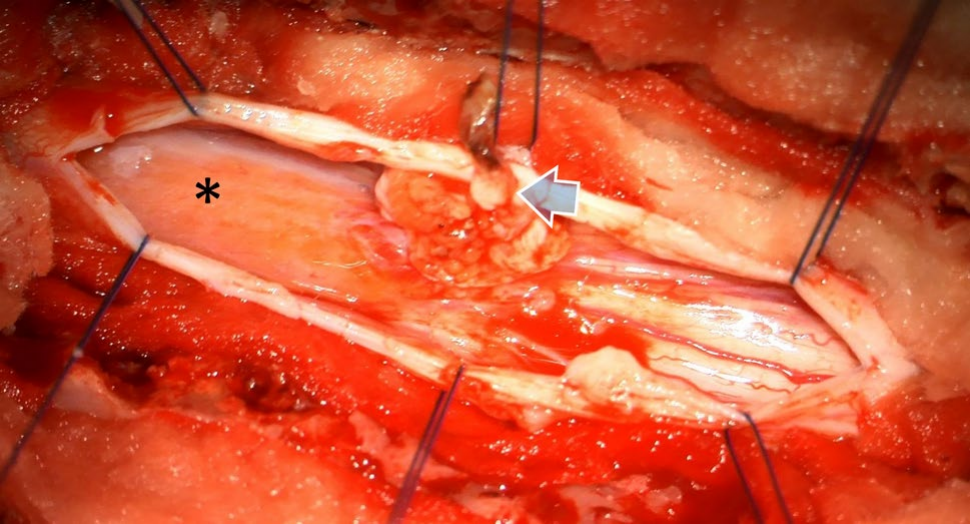
Intradural operative view: after dural opening, visualization of the fibrous tract (*arrow*) attachment to the spinal cord and the associated intradural lipoma (*asterisk*) prior to detethering and partial resection

A subperiosteal dissection of the spinous processes and laminae from T2 to T5 was performed. Fusion of the laminae was observed at the T4 level, with a canal for the fibrous tract crossing through the T4 spinous, process and extending towards the dura mater (Figs. 3a, b and 4).

**Fig. 5:**
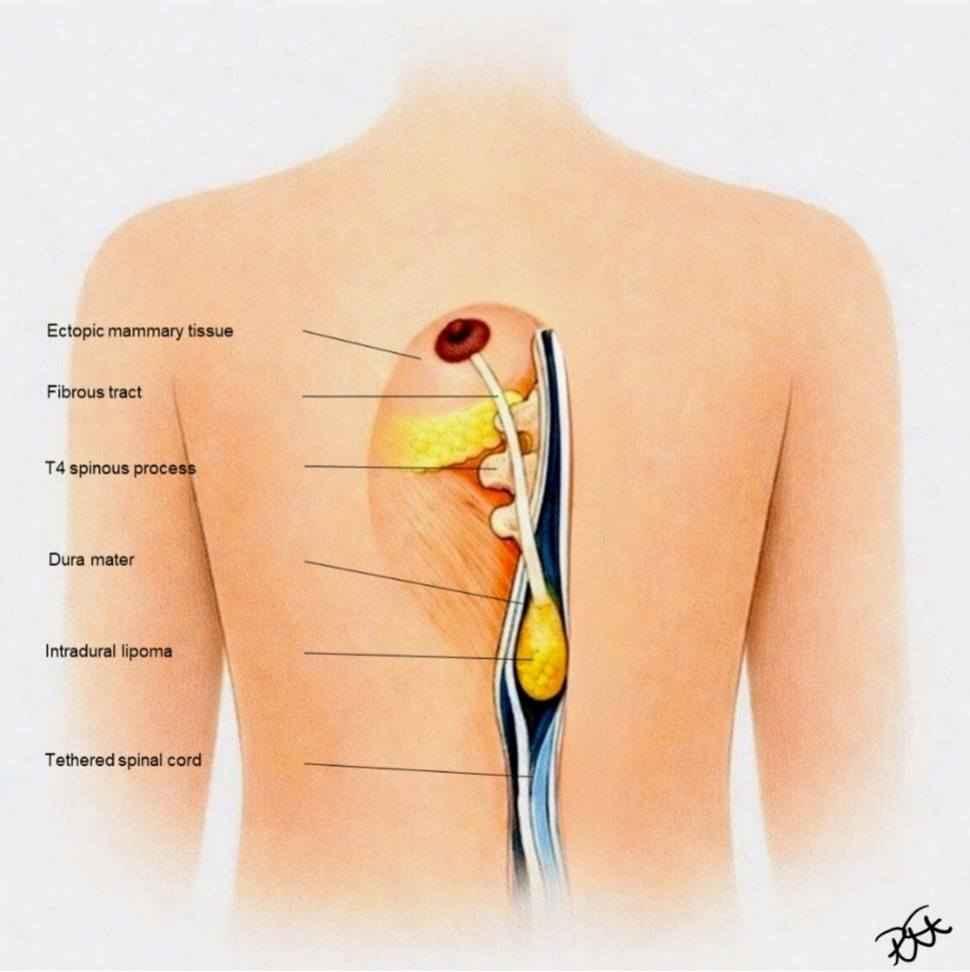
Schematic illustration showing the anatomical relationship between the dorsal ectopic mammary tissue and the tethered spinal cord, shown from superficial to deep planes. A fibrous tract originates from the dorsal ectopic mammary tissue, which extends towards the spine, going through the T4 spinous process before entering the spinal canal. The tract continues to the dura mater and connects with the intradural lipoma, resulting in tethering of the thoracic spinal cord. Hand-drawn illustration by P.A. edited in Windows Surface Pro ® and PowerPoint®

Following dural opening, the end of the fibrous tract attached to the spinal cord was visualized, along with an intradural lipoma extending cranially (Fig. 4). The tract was detached, and resection of the lipoma was performed to achieve adequate spinal cord detethering (Fig. 5).

The dura mater was closed primarily, and the laminae were repositioned. Muscular and fascial layers were closed in anatomical planes. Additional subcutaneous adipose tissue was removed, and the skin was closed using an intradermal running suture.

**Fig. 6:**
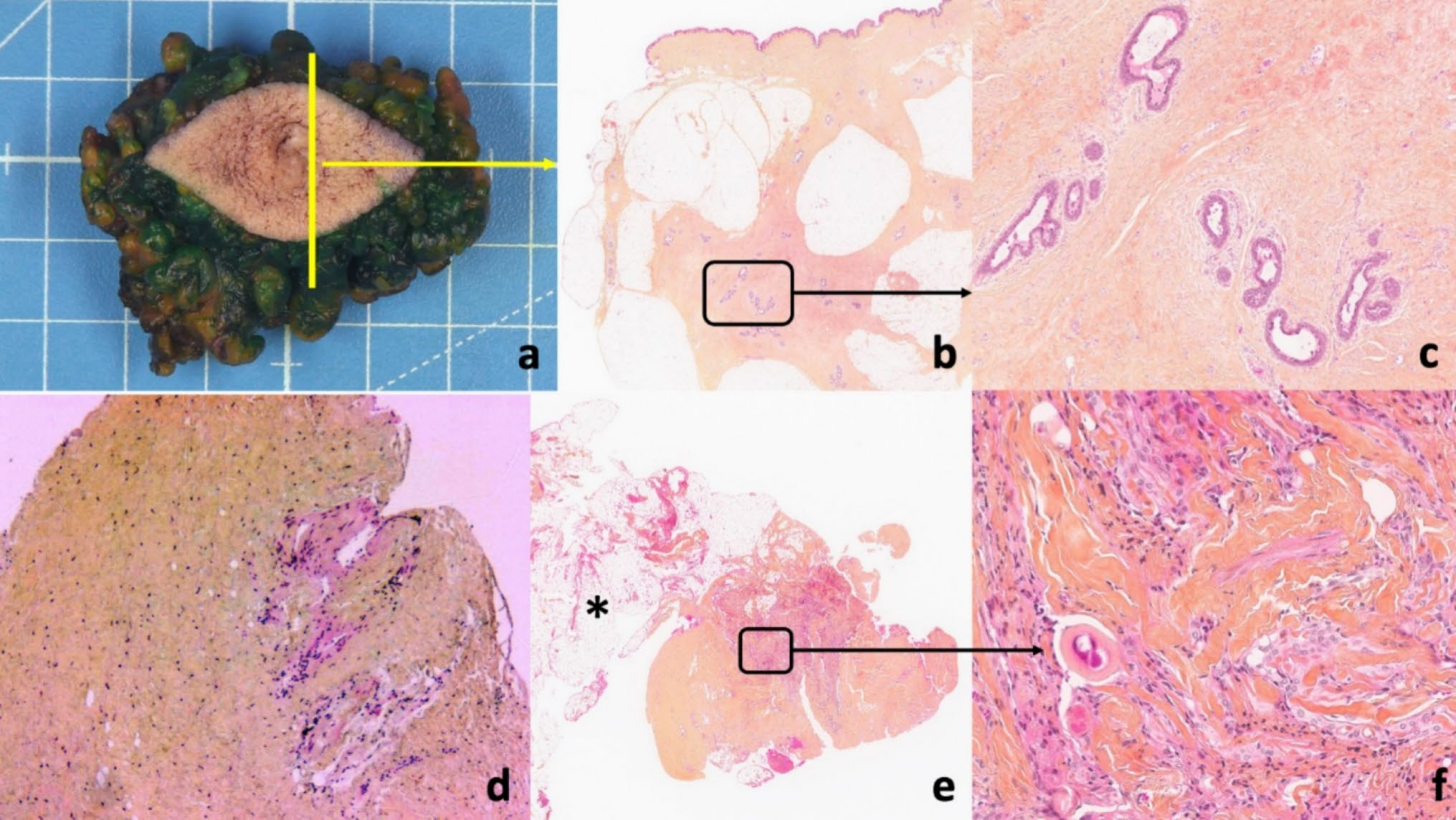
Pathological findings: **a–c** Ectopic mammary tissue: **a** macroscopic appearance; **b** histological section showing a fibro-adipose tissue with a few lactiferous ducts (**c** high-magnification view × 50). **d** Tract connecting the nipple to the dura mater: fibrous tissue containing blood vessels. **e** Extramedullary lipomatous adipose tissue (*asterisk*) attached to fibrous meninges with hyperplastic meningothelial cells and calcifications (**f** high-magnification view × 200)

Histopathological examination of the excised specimen confirmed the diagnosis of ectopic mammary tissue. Microscopy demonstrated mammary glandular elements within fibro-adipose tissue, including scattered lactiferous ducts. The tract extending from the nipple to the dura mater was composed of vascularized fibrous tissue. Additionally, extramedullary lipomatous adipose tissue attached to fibrous meninges, accompanied by meningothelial hyperplasia and calcifications, was observed, consistent with the radiological diagnosis of an intradural lipoma (Fig. 6).

Postoperative recovery was uneventful, with no neurological deficit or surgical complication. The patient remained neurologically intact on follow-up.

## Prognosis

Supernumerary mammary structures are relatively common congenital anomalies resulting from incomplete regression of the embryonic mammary ridge [2]. These structures typically occur along the milk line, particularly in the axillary or inframammary regions. In contrast, ectopic mammary tissue outside this line is uncommon, and dorsal localizations are exceptionally rare [6]. Reported locations include the face, shoulder, arm, thigh, vulva, and the back. Because dorsal lesions may clinically resemble lipomas, dermoid cysts or other benign soft- tissue masses, the diagnosis may initially be overlooked [5].

Dorsal midline cutaneous lesions are of particular clinical importance because they may represent markers of occult spinal dysraphism, a spectrum of congenital anomalies resulting from abnormal neural tube closure. Several cutaneous stigmata have been described in association with occult spinal dysraphism, including dermal sinus tracts, lipomas, hemangiomas, hypertrichosis, skin appendages, and subcutaneous masses. Among these conditions, spinal lipomas are a common cause of tethered cord syndrome, in which abnormal attachments restrict spinal cord mobility [7]. As a child grows, progressive traction on the cord may lead to neurological deterioration, including motor weakness, sensory disturbances, orthopaedic deformities, and bladder dysfunction [8].

**Fig. 7:**
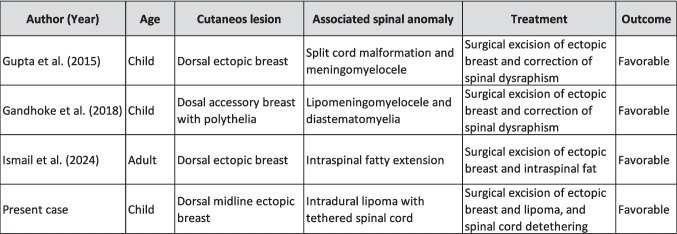
Reported cases of dorsal ectopic mammary tissue associated with spinal dysraphism. The present case illustrates a rare association between dorsal ectopic mammary tissue and intradural lipoma with tethered cord

The association between ectopic mammary tissue and spinal dysraphism is extremely rare. Only a limited number of cases describing dorsal ectopic mammary tissue associated with spinal anomalies have been reported (Fig. 7). Gupta et al. described a dorsal ectopic breast associated with split cord malformation and meningocele, suggesting a possible link between ectopic mammary tissue and neural tube defects [9]. Similarly, Gandhoke et al. reported a case of dorsal accessory breast with polythelia associated with complex spinal dysraphism, including lipomeningomyelocele and diastematomyelia [10]. More recently, Ismail et al. described a dorsal ectopic breast with intraspinal extension containing fatty components [6]. These reports highlight that unusual dorsal mammary structures may occasionally represent cutaneous markers of underlying spinal anomalies.

In the present case, MRI demonstrated an intradural lipoma connected to the cutaneous lesion by a fibrous tract, resulting in tethering of the spinal cord (Fig. 2). The presence of this direct anatomical connection supports the hypothesis that certain dorsal mammary anomalies may represent cutaneous manifestations of spinal dysraphism (Fig. 5).

The coexistence of dorsal ectopic mammary tissue and spinal dysraphism may be explained by disturbances early during embryologic development. The mammary ridges arise from ectodermal thickenings during the sixth week of gestation, extending from the axillary to the inguinal regions [2]. Normally, these ridges regress except in the pectoral region where the definitive mammary glands develop. Persistence or aberrant migration of mammary ridge elements may result in accessory nipples or ectopic breast tissue [5].

During the same developmental period, the neural tube undergoes closure and separation from the surface ectoderm, a process known as disjunction [1]. Abnormal interactions between the developing neural tube, mesenchyme, and surface ectoderm may result in forms of spinal dysraphism, including lipomas and tethered cord.

Defective disjunction may also allow persistent connections between cutaneous structures and the spinal canal through fibrous tracts or lipomatous tissue, as observed in the present case. The association of ectopic mammary tissue with spinal dysraphism may therefore reflect a shared ectodermal developmental disturbance, although the precise mechanism remains uncertain due to the rarity of reported cases.

Management of tethered cord syndrome in children is primarily surgical and aims to release the spinal cord to prevent progressive neurological deterioration. Even in asymptomatic patients, prophylactic detethering is generally recommended when imaging demonstrates clear anatomical tethering. In our case, the combined surgical approach allowed simultaneous excision of the ectopic mammary tissue and release of the tethered cord, with an uneventful postoperative course (Fig. 5).

This case highlights the importance of careful evaluation of congenital dorsal cutaneous lesions. Recognition of atypical dorsal mammary structures should prompt consideration of spinal imaging, as these lesions may represent previously underrecognized cutaneous markers of spinal dysraphism requiring neurosurgical assessment.

Spinal MRI remains the imaging modality of choice for evaluating suspected dysraphism and identifying associated abnormalities such as lipomas, tethered cord, or fibrous tracts [11]. Early identification allows timely neurosurgical management and may reduce the risk of long-term neurological complications.

## Outcomes

Dorsal midline ectopic mammary tissue is an exceptionally rare congenital anomaly. When located in the midline of the back, it may represent a cutaneous marker of underlying spinal dysraphism, including intradural lipoma and tethered cord.

Careful clinical examination and spinal MRI should always be carried out in such cases. Early multidisciplinary management allows appropriate surgical treatment and may prevent long-term neurological complications.

## Data Availability

No datasets were generated or analysed during the current study.
